# Novel Populations of Lung Capillary Endothelial Cells and Their Functional Significance

**DOI:** 10.21203/rs.3.rs-2887159/v1

**Published:** 2023-05-03

**Authors:** Joel James, Aleksandr Dekan, Maki Niihori, Nolan McClain, Mathews Varghese, Dinesh Bharti, Odunayo Susan Lawal, Marco Padilla-Rodrigez, Dan Yi, Zhiyu Dai, Oleg Gusev, Olga Rafikova, Ruslan Rafikov

**Affiliations:** University of Arizona; University of Arizona; University of Arizona; University of Arizona; University of Arizona; University of Arizona; University of Arizona; University of Arizona; University of Arizona; University of Arizona; Juntendo University; University of Arizona; University of Arizona

**Keywords:** general capillary, endothelium, single cell analysis, microcirculation, pulmonary circulation

## Abstract

The role of the lung’s microcirculation and capillary endothelial cells in normal physiology and the pathobiology of pulmonary diseases is unequivocally vital. The recent discovery of molecularly distinct aerocytes and general capillary (gCaps) endothelial cells by single-cell transcriptomics (scRNAseq) advanced the field in understanding microcirculatory milieu and cellular communications. However, increasing evidence from different groups indicated the possibility of more heterogenic structures of lung capillaries. Therefore, we investigated enriched lung endothelial cells by scRNAseq and identified five novel populations of gCaps with distinct molecular signatures and roles. Our analysis suggests that two populations of gCaps that express Scn7a(Na^+^) and Clic4(Cl^−^) ion transporters form the arterial-to-vein zonation and establish the capillary barrier. We also discovered and named mitotically-active “root” cells (Flot1+) on the interface between arterial, Scn7a+, and Clic4 + endothelium, responsible for the regeneration and repair of the adjacent endothelial populations. Furthermore, the transition of gCaps to a vein requires a venous-capillary endothelium expressing Lingo2. Finally, gCaps detached from the zonation represent a high level of Fabp4, other metabolically active genes, and tip-cell markers showing angiogenesis-regulating capacity. The discovery of these populations will translate into a better understanding of the involvement of capillary phenotypes and their communications in lung disease pathogenesis.

## Introduction

Despite extensive research spanning multiple decades, the field of pulmonary vascular diseases continues to face a scarcity of effective therapeutic options. One contributing factor to this challenge is the limited understanding of lung vascular biology, particularly the heterogeneous nature of endothelial cells (EC) and the intricate interactions among cells within pulmonary circulation and beyond. A recent discovery of heterogeneity in lung capillary cells offers a critical insight into the organization of capillary networks and cell-cell communications within the microvasculature^1^. Indeed, the knowledge of two distinct endothelial populations – aerocytes and general capillary cells (gCaps) - could reshape the pulmonary field due to vast differences between these endothelial cell types previously attributed to a single type of microvascular EC.

Capillary ECs orchestrate numerous essential pulmonary vascular functions, including maintaining and repairing the capillary network, facilitating angiogenesis, ensuring blood barrier function, and enabling gas and nutrient exchange^2,3^. They also engage in a complex network of interactions with various cell types, such as inflammatory cells, platelets, fibroblasts, pericytes, smooth muscle, and epithelial cells. Understanding specialized endothelial types enables more comprehensive navigation of this intricate system than analyzing averaged phenotypes. Recent advantages in single-cell transcriptomics analysis have significantly aided this research direction. The data from many research groups based mainly on whole lungs have indicated that the capillary cells are more heterogeneous and potentially feature additional phenotypes beyond the described aerocytes and gCaps^4,5^. Yet, resolving the capillary endothelium remains a challenge. In this study, we employed highly enriched endothelial cells from rat lungs to explore the heterogeneity of capillary cells with unprecedented resolution. Our investigation resulted in the significant discovery of five distinct phenotypes of gCaps, each exhibiting unique specializations.

## Results

### Organization of the lung endothelium from single-cell data

**Figure 1:**
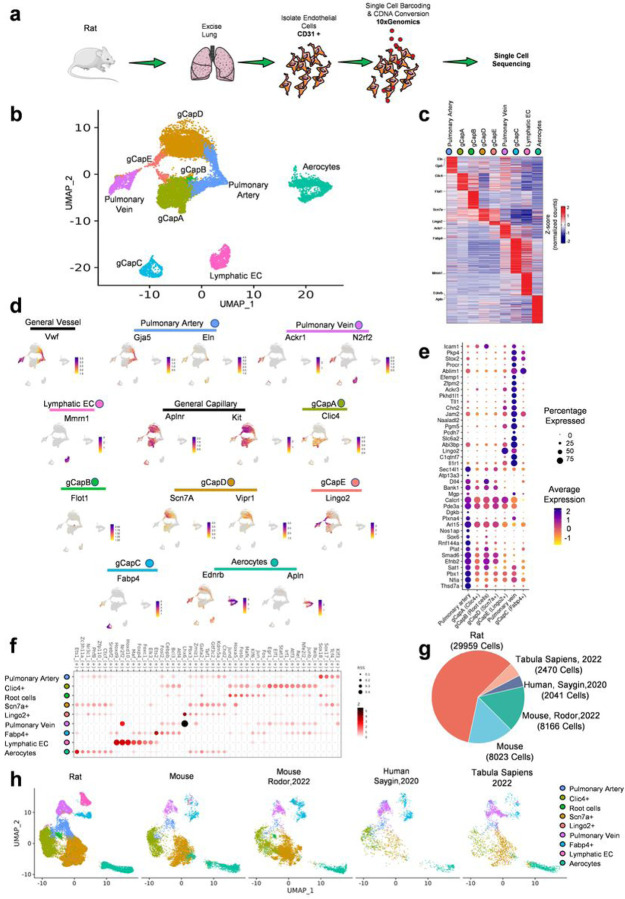
**a** Study design showing rat lung endothelial isolation and 10x barcoding. **b** UMAP plot showing arterial-to-vein zonation of endothelial populations in two female rat samples. **c** Heat map visualizing conserved differentially expressed that differentiate endothelial populations in two female and two male samples. **d** UMAP plots showing gene expression of cell type markers. Scales for Vwf, Eln, Clic4, Flot1, Scn7a, Vipr1 have gene expression cut-offs to highlight regions with high expression. **e** Dot plot showing the top 40 genes that differentiate pulmonary vein and artery. General capillary phenotypes are arranged along the artery-to-vein zonation (adjusted p-value < 0.05, Two-sided Wilcoxon rank sum test with Bonferroni correction). **f** Heat map visualizing regulon specificity scores of endothelial phenotypes. **g** Pie chart showing the number of cells in each dataset used for cross-species integration. **h** UMAP-plots showing cross-species integration and cell annotations transferred from rat data.

To improve endothelial cell viability and enrichment for high-resolution analysis, we isolated CD31-positive cells from rat lungs using anti-CD31 antibody and magnetic beads (Fig. 1a), followed by processing with 10x genomics single-cell RNA sequencing (scRNAseq) (Supplementary data Fig. 1a,b). After mapping and annotating isolated ECs on the UMAP plot (Fig. 1b, Supplementary data Fig. 1c-g), we identified five novel populations, termed gCaps A-E. The lineage of these newly discovered ECs was determined based on the expression of the Apelin receptor, a previously reported gCaps marker^1^. Notably, novel gCaps demonstrated the formation of two unique zonations between pulmonary artery and vein ECs (AV-zonation) involving gCaps A, B, D, and E phenotypes (Fig. 1b). This finding suggests a gradual phenotypic transition in capillary cells between arterial and vein ECs. Each cell type expressed a set of markers, as shown in the heatmap (Fig. 1c), highlighting the uniqueness of the identified cell types. To annotate cells and AV zonation, we utilized various markers based on the most distinguishing features. By employing gene expression cut-offs to emphasize regions of high expression, we effectively illustrated the phenotype transition along AV-zonation in Fig. 1d. The macrovascular ECs from the pulmonary artery and vein express the Von-Willibrant factor (Vwf). The endothelial cell cluster block began at the converging right corner of the two AV zonations, with Elastin (Eln) expression required in a high-pressure environment in mature arterial ECs from large pulmonary arteries^6^. Pulmonary artery ECs continued from this corner, marked by the previously established gap junction gene Gja5, and formed two zonation arms transitioning into general capillary ECs (Kit, AplnR positive). Ackr1 and N2rf2 gene expressions visualized pulmonary vein ECs at the left corner of the endothelial clusters in zonation. The lymphatic (Mmm1+) and aerocyte (Ednrb+, Apln+) ECs showed separate clusters outside of AV zonation, implying the distinct and highly specialized phenotypes for these endothelial cells not associated with the endothelial transition from artery to vein (Supplementary Fig. h).

### Novel populations of capillary cells general

Our data indicated five novel phenotypes of what was previously considered a single general capillary cell phenotype (Fig. 1d). Two large clusters of gCaps form the lower arm with gCapA with the highest expression of the chloride channel (Clic4+), and the upper arm, gCapD, highly expressing the sodium channel (Scn7a+). The zonations converge with the venous-capillary gCapE (Lingo2+), which connects to the pulmonary vein ECs. Consequently, gCapsA, D, and E create a continuous capillary zonation from the artery to the vein. A small yet persistent subset of gCapB (Flot1+) is situated at the interface between pulmonary artery cells and gCapsA&D, also on (Supplementary File 1, 3D UMAP). RNA velocity analysis identifies this cell type as the “root” or origin for gCapsA&D and arterial ECs (Supplementary data Fig. 1i,i’). Due to the highly mitotic nature of these cells and activation of transcriptional factors involved in EC proliferations such as Jun and Fos^7^ (Supplementary data Fig. 1f,j), we hypothesize that gCapsB may play a role in repairing capillary ECs and contribute to capillary rejuvenation and neo-capillary genesis. Finally, gCapsC displays distinct separation of the cluster outside of the AV-zonation, highly expressing Fabp4. Comparing the new gCaps with arterial and venous endothelial cells revealed similarities between gCapsA(Clic4+), B(Root), and D(Scn7a+) with arterial ECs, and gCapE(Lingo2+) with venous ECs (Fig. 1e). In contrast, gCap C exhibited a completely different expression pattern. We used SCENIC to calculate regulon specificity scores, which show (Fig. 1f, Supplementary data Fig. 1j) that the novel phenotypes of gCaps have different transcriptional programs, representing distinct cell populations. We also build UMAP using the activity of transcription factors (Supplementary data Fig. 1j,k) that resembles the structure of a gene-based UMAP on principal components (Fig. 1b). Finally, we found the top 5 most specific transcription factors for each phenotype using regulon specificity scores (RSS) (Supplementary data Fig. 1l). We didn’t observe a distinct separation of capillary cells in publicly available human or mouse datasets. Thus, we also added our mouse dataset into cross-species integration, in which cells were isolated by FACS using endothelial expression of GFP (Tg(TIE2GFP)287Sato/J mouse). We achieved a three-fold higher resolution for rat endothelial cells (30k cells) compared to mouse sets (8k)^8^ or human sets (2k) data^9,10^ (Fig. 1g). We analyzed and visualized the integrated data (Fig. 1h), including cell type markers. Notably, the publicly accessible human or mouse datasets possess no clear distinction of pulmonary capillary cells. In particular, human datasets lacked capillary markers Aplnr and Kit but had high Vwf, Eln, Gja5, Nr2f2 expressions related to large vessels ensothelial cells, indicating a marked loss of capillary cells^9,10^ (Supplementary data Fig. 2a). This effect can be attributed to the harvesting of cells from post-mortem lung tissues and the preparation process for whole lung sequencing, which is associated with a significant loss of viable capillary cells. In contrast, the publicly available mouse dataset was enriched with capillary cells, but only showed a partial separation of cellular phenotypes^8^. In addition, the expression of cell type markers in publicly available mouse data was weak compared to our mouse dataset. Incorporating our mouse dataset collected from mice expressing endothelial GFP, which allowed FACS-based endothelial enrichment, enhanced a cross-species integration. We showed that our isolated mouse and rat endothelial cells had a better quality in terms of the number Unique molecular identifier counts (UMI counts) and/or genes detected in each cell (Supplementary data Fig. 2b). This allowed us to successfully identify the newly described phenotypes of gCaps in mouse datasets in addtion to rats. We also found a significant intersection of cell markers among our mouse and rat datasets (Supplementary Table 2).

### Characteristics of novel general capillary endothelial cells gCapC (Fabp4+)

**Figure 2:**
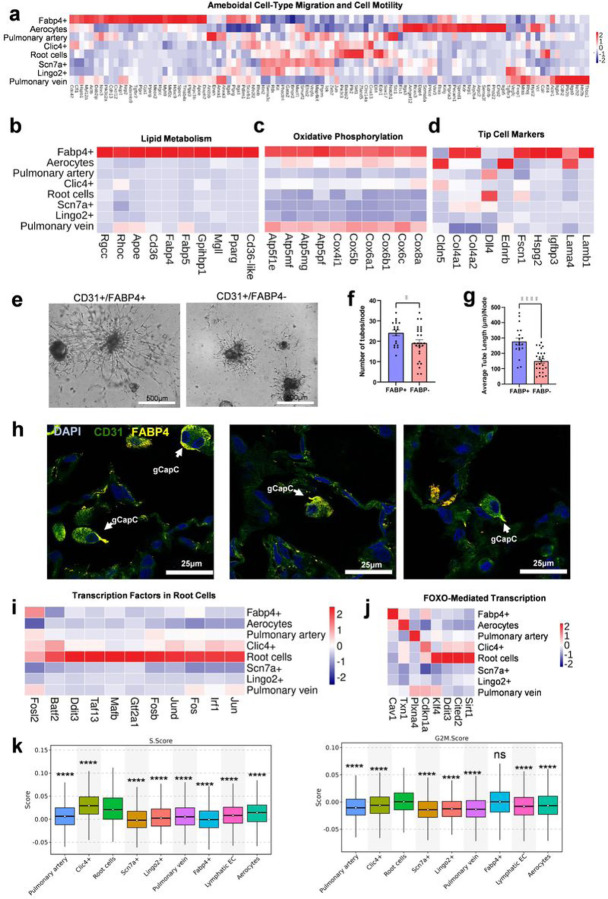
**a-d**, **j** Heat maps showing the expression of conserved differentially expressed genes that differentiate cell types present on the plot by groups. **a** Merged genes from KEGG cell motility and Gene Ontology ameboidal-type cell migration. **b** Selected lipid metabolism genes. **c** Oxidative Phosphorylation genes from KEGG. **d** Tip Cell Markers^41^. **e** CD31+ and FABP4+ cells seeded on a matrigel form tube sprouting spheroids. CD31+ and FABP4- cells show impaired tube formation. **f** Quantification of tubes show depletion of FABP4+ cells impair number of tubes (*p< 0.05, t-test). **g** FABP4 depleted spheroids have stunted tube growth as indicated by tube length (****p< 0.0001, t-test). **h** Confocal imaging showing FABP4+ cells in the lung. **i** Root-cells-conserved transcription factors expression. **j** Genes from Reactome FOXO-mediated transcription. **k** Cell Cycle Phase S and G2M scores compared to Root cells (**** p< 0.0001, two-sided Wilcoxon rank sum test).

Fabp4 + gCaps form a distinct cluster on the UMAP, resembling lymphatic ECs or aerocytes clusters rather than being present at the AV zonation. This observation implies their increased specialization and perhaps indicates a reduced structural participation in the capillary network. The Fabp4 + cells classify as gCaps because they express Aplnr, Cdh5, Myo10, and Ccdc85a, along with other phenotypes of gCaps (Fig. 1d). Further analysis shows that Fabp4 + cells exhibit a high enrichment of genes responsible for ameboid-type migration and cell motility (Fig. 2a, Supplementary Table 3). These cells also express genes involved in lipid metabolism (Pparg, CD36, Fabp4/5) and oxidative phosphorylation (Fig. 2b,c), indicating their high energy demand compared to other endothelial cells. This finding suggests that these cells rely on lipid-centered metabolism for their angiogenic capabilities. Previous studies have demonstrated that angiogenesis depends on lipid metabolism and is primarily impaired in Fabp-deficient mice^11^. A comparison of the gene list for tip-cell markers revealed that most tip-cell markers are highly expressed in Fabp4 + capillary cells (Fig. 2d). Tip ECs play a crucial role in angiogenesis, guiding the sprouting of new capillaries/vessels and facilitating branching angiogenesis^12^. Our study, for the first time, identifies a specific cell population within gCaps in the lung responsible for capillary angiogenesis. These cells are also rendered in published mouse data and humans (Supplementary data Fig. 2a). However, Fabp4 + cells are sometimes mistaken for macrophages due to their high motility and expression of various cytokines. Nevertheless, Fabp4 + cells do not express macrophage markers such as CD68 (Supplementary data Fig. 3a. Supplementary Table 4, 5). This study contributes to the annotation of this particular phenotype of gCaps. To examine the angiogenic properties of Fabp4 + cells, we employed cell sorting to isolate CD31 + cells and CD31 + cells lacking Fabp4 + cells (Supplementary data Fig. 3b). The sprouting experiment conducted on matrigel revealed a decreased angiogenic capacity in Fabp4 + population deficient CD31 + cells (Fig. 2e–g). Fluorescent images displayed distinct features of these cells, including lamellipodia-like structures (Fig. 2h, Supplementary data Fig. 3c).

### gCapB (Root/Flot1+)

A minor population of gCapB (Flot1+) root cells is situated within the AV-zonation at the interface of three cell types: small artery endothelial cells, gCapA (Clic4+), and gCapD (Scn7a+) (Fig. 1b, d, Supplementary data Fig. 1i, i’). We deduced root and end cells from the velocity graph using a Markov chain implemented in CellRank, and identified several root states (Supplementary Fig. 1i Root and End cells), with gCapB among the root cells. The partition-based graph abstraction (PAGA) analysis, enhanced by velocityinferred directionality, revealed a high transcriptional similarity between gCapB cells and Scn7a+, Clic4+, Lingo2+, and pulmonary artery cells, which exhibited less similarity to each other than to gCapB (Supplementary Fig. 1.i’). Moreover, gCapB cells appear to be the principal root cells, as all other cells, except for pulmonary veins, are descendants of them based on directed transition probabilities. Consequently, we hypothesize that gCapB cells serve as the primary root cells responsible for regeneration. Our data indicate several important transcriptional factors (TFs) are highy expressed in Root cells, such as Fos and Jun, members of the AP-1 family. These essential transcription factors promote endothelial cell proliferation and angiogenesis (Fig. 2i)^13^. Conversely, the Foxo transcription factor and Foxo pathways (Fig. 2j) were upregulated in Root cells. The Foxo pathway is a crucial regulator of cellular homeostasis, playing a role in cell cycle regulation, apoptosis, and oxidative stress responses^14^. In endothelial cells, Foxo1 regulates angiogenesis by controlling the expression of VEGF and other angiogenic factors. Additionally, Foxo1 has been implicated in regulating endothelial cell migration, a critical step in angiogenesis^15^. Overall, Fos, Jun, and Foxo transcription factors are essential regulators of endothelial cell proliferation and angiogenesis, and their dysregulation can contribute to the development of various angiogenic diseases. Thus, the Root cells may play a central role in regulating capillary and arteriole formation and repair by being the source of endothelial cells. Indeed, the Root cells showed a high G2M and S score; the G2M score is a critical marker for indicating mitotically active cells (Fig. 2k). This suggests the role of Root cells as the origin of capillary and pulmonary artery endothelium. Our data also showed high expression of CDKn1a,1c (p21, and p57) isoforms (Fig. 2j) is necessary for stem cell maintenance and differentiation to harness high proliferation rate.

### gCapA (Clic4+) and gCapD (Scn7a+)

**Figure 3:**
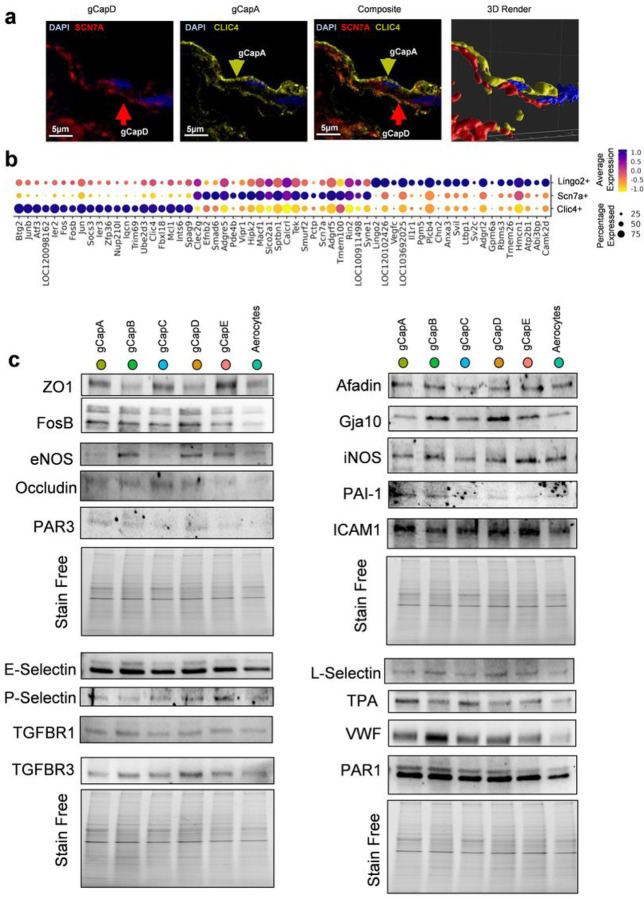
**a** Confocal imaging shows the alignment of gCapA and gCapD in the lungs. **b** Dot plot showing the top 60 genes that differentiate each of the general capillary phenotypes (adjusted p-value < 0.05, Two-sided Wilcoxon rank sum test with Bonferroni correction). **c** Western Blots of gCaps A-E and Aerocytes showing ZO-1(Zonula occludens-1), FosB (FosB Proto-Oncogene, AP-1 Transcription Factor Subunit), eNOS(endothelial Nitric oxide synthase), Occludin, PAR3 (Proteinase-activated receptor 3), E-Selectin, P-Selectin, TGFBR1 (transforming growth factor-beta (TGF-β) receptor type 1), TGFBR3 (transforming growth factor-beta (TGF-β) receptor type 3), Afadin, Gja10 (Gap Junction Protein Alpha 10), iNOS (inducible nitric oxide synthase), PAI-1(plasminogen activator inhibitor 1), ICAM1 (Intercellular Adhesion Molecule 1), L-Selectin, TPA (Tissue plasminogen activator), VWF (von Willebrand factor), PAR1 (proteinase-activated receptor 1)

Clic4 + and Scn7a + cells constitute the two primary populations of gCaps, which play a significant role in constructing the capillary network around the alveoli to facilitate gas transport throughout the organism. These endothelial types are present in two distinct AV-zonations, connecting a small artery to vein endothelium via gCapE (Lingo2+) (Fig. 1d). This raises two critical questions: First, do they form distinct capillaries? Second, what unique roles does each type play? Our confocal fluorescent imaging indicates that Scn7a + and Clic4 + cells can form extensive capillary structures with both phenotypes, particularly around aerocytes (Fig. 3a, Supplementary data Fig. 4a,b).

Numerous genes in Clic4 + cells within the top 50 differentially expressed regulate barrier maintenance, function inflammatory cell and ion/fluid trafficking, inflammation, and coagulation control (Supplementary Table 1). Analysis of differentially expressed genes indicates that Clic4 + cells actively regulate cell growth control through upregulation of Bgt2 and Socs3. Elevated expression of Atf3 controls the metabolism of Clic4 + cells and has been implicated in endothelial cell activation.

Conversely, Scn7a + cells exhibit a greater representation of hormonal/soluble factors-based regulation through various receptors. They display highly expressed Vipr1 and Npr3 receptors, which regulate vasodilation^16^. The Adgr family of G-protein coupled receptors, including CD97, Latrophillin, and Gpr116, are involved in cell-cell interactions, cell adhesion, and migration (Fig. 3b)^17^. Ephrin-B2 and Calcrl receptors modulate angiogenesis and cell adhesion^18,19^. Scn7a + cells also express receptors responsible for proliferation and survival, such as c-Kit, Tie2, Bmpr2, Lifr, and co-receptor Eng (Fig. 3b). Lastly, two tyrosine phosphatase receptors, Ptprm and Ptprb, and integrin Itga1, play roles in cell adhesion and maintaining barrier function^20^. Therefore, we may conclude that transcriptional regulators predominantly program Clic4 + cells, while Scn7a + cells are primarily controlled by paracrine/autocrine signaling via numerous receptors. Further clarification regarding the differences between these cell types can be found in Fig. 2k, which illustrates the highly mitotically active Clic4 + cells alongside the much less active Scn7a + cells. This suggests that these cell types represent different stages of capillary cell maturation.

Intriguing is that one capillary population (Scn7a+) mainly expresses a sodium transporter, whereas another population (Clic4+) highly expresses the chloride channel. Both channels are essential for controlling the exchange of molecules (ions, solutes, and water) between the blood and the surrounding lung tissue, which is the primary physiological role of capillaries. Moreover, these channels can maintain the electrochemical gradient leading to membrane polarization and, perhaps, promote a tighter barrier.

### gCapE (Lingo2+)

Lingo2 + cells are situated in the AV zonation between two gCaps (Clic4 + and Scn7a+) types and venous ECs, which characterizes them as venous-capillary cells. These cells express unique genes, primarily indicative of capillary endothelial characteristics. Firstly, Lingo2 + cells express the Lingo2 gene, which is involved in the Nogo signaling pathway^21^. Although Nogo signaling mostly pertains to neuronal system development and maintenance, NogoA/B has been reported to contribute to angiogenesis and the inflammatory response in endothelial cells^22,23^. Consequently, unusually high Lingo2 expression may suggest these cells participate in angiogenesis and chemokine response. Furthermore, Lingo2 + cells express the Ackr3 (Cxcr7) receptor, unlike venous ECs, which predominantly represent Ackr1. Ackr3 specifically interacts with Cxcl12^24^, modulating angiogenesis, while venous Ackr1 scavenges all CC and CxC chemokines, aiding inflammation regulation^25^.

Endothelial cells perform various functions, such as maintaining barrier integrity, vasodilation, inflammation, and angiogenesis^26^. Our study identified a high degree of heterogeneity among capillary cells, prompting us to investigate whether proteins with essential roles are differentially expressed across endothelial cell types, reflecting a higher level of specialization in contrast to their previously assumed shared functionality among endothelial cells. We employed Western blot analysis to assess protein levels in the EC populations (Fig. 3c). Our findings revealed considerable functional heterogeneity among these endothelial cell types, supporting the notion of specialized endothelial functions within distinct populations^27^.

### Interactions between populations of endothelial cells

Pulmonary circulation is a highly specialized vascular network, and its proper functioning depends on the coordinated interplay among various cellular components. These components include endothelial cells, smooth muscle cells, pericytes, fibroblasts, and inflammatory cells, which interact via cell-cell communication and local oaracrine signaling. Endothelial cell interactions are crucial in forming and stabilizing new blood vessels during angiogenesis and establishing the blood-tissue barrier^28^. Throughout this process, endothelial cells communicate using various signaling molecules to regulate cell adhesion, migration, and proliferation^29^. Understanding the heterogeneity in endothelial cells can help us analyze cell-cell interactions in more detail.

**Figure 4:**
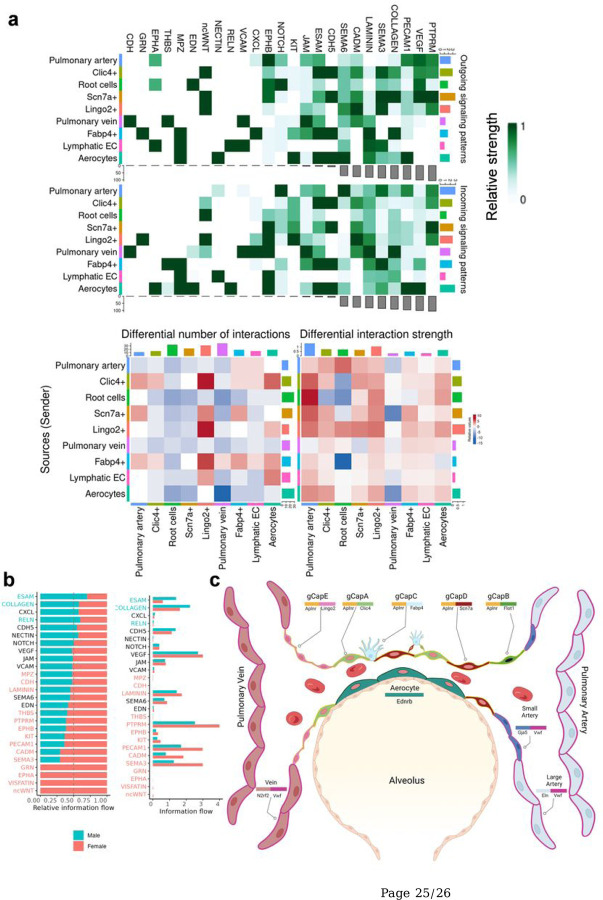
**a** All outgoing and incoming signaling pathways in endothelial populations on the aggregated cell-cell communication network from 2 female and 2 male rats. **b** Information flow comparison of signaling pathways in males and females. Two-sided Wilcoxon rank sum test with Bonferroni correction (adjusted p-value < 0.05). **c** Pulmonary capillaries consist of distinct types of gCaps and aerocytes, each with specific functions. Aerocytes, located at the alveolar epithelium interface, play a crucial role in gas transport. The primary gCaps, Clic4+ and Scn7a+, form the intricate capillary network. Meanwhile, gCapB, or root cells, are situated at the interface between small arteries and gCaps A/D, contributing to regeneration and repair processes. gCapC, represent tip-like cells and participate in angiogenesis. Lastly, gCapE cells connect capillaries with veins.

The most significant communication signals emerge from Scn7a + cells in paracrine and autocrine manners (Fig. 4a). Clic4 + cells, aerocytes, and pulmonary artery cells also substantially contribute to cell-cell interactions in the lungs. Additionally, several intriguing cellular communications, suggesting novel phenotypes, are discovered. For instance, Scn7a + cells communicate with aerocytes using Sema3 and pulmonary arteries using Sema6 for vessel guidance and cellular signaling. The angiogenic chemokine Cxcl12 exhibits outbound signaling from Fabp4 + and arterial ECs. Proliferating c-kit signaling initiates from aerocytes and targets two primary gCaps: Scn7a + and Clic4+. Interestingly, Root cells play a vital role in endothelin signaling to aerocytes. All outgoing and incoming signaling patterns are illustrated in Fig. 4a, while the complete connectome analysis can be found in supplementary Fig. 5.

### Sex difference

The overall connectome analysis revealed that Esam and Reelin (Reln) signaling are preferentially upregulated in males, while non-canonical WNT, Visfatin (Nampt), Progranulin (Grn), EphA/B, and Semaphorin 3 are upregulated in females (Fig. 4b). As a result, the Reelin and Visfatin systems appear to regulate the inflammatory response in a sex-dependent manner. Furthermore, Esam and Ephrins (EphA/B) modulate sex-dependent adhesion to endothelial cells^18^. Females also possess two pro-survival systems, non-canonical WNT and GRN, which regulate vascular cell proliferation and repair. Sex-specific information about connectome for the different cell type and differential expression genes are available in supplementary Fig. 6–8 and supplementary table 6.

## Discussion

Endothelial functionality and cell-cell interactions are vital for maintaining the integrity and function of pulmonary circulation. In this study, we reveal that the previously described gCaps are composed of several distinct cell populations, each with unique specializations. These range from blood-tissue barrier formation (gCaps A&D) to regeneration (gCap B) and angiogenesis (gCapC) (Fig. 4c). Strikingly, we used two different approaches to investigate capillary cell types, which included two species and two isolation methods. The first approach involved using magnetic beads to enrich CD31 + cells from rats, followed by scRNA analysis. The second approach utilized a genetically modified mouse model which expressed GFP in endothelial cells, sorted using FACS. Both methods of isolation and different models provided comparable results, underscoring the robustness of our discovery.

These cells display intriguing new patterns of cell-cell interactions. Disruptions in these interactions and the functionality of these phenotypes can lead to a variety of pulmonary vascular diseases, such as pulmonary hypertension, acute lung injury, and chronic obstructive pulmonary disease (COPD). This discovery has the potential to revolutionize vascular research by challenging the notion that microvascular cells are a single cell type, as we now understand that they are a complex mixture of signals from various cells^30^. It is possible that other organs harbor similar sets of gCaps, such as Fabp4 +^31^ or root cells.

Unfortunately, our current treatment options targeting pulmonary vasculature are inadequate, a reality that became utterly obvious during the SARS-CoV-2 pandemic. Gaining a deeper understanding of the role of each endothelial cell type in the capillary could vastly improve our knowledge of microvasculature function, signaling pathways, secretomes, and cell-cell interactions. This newfound insight could ultimately pave the way for the development of more targeted therapies for a wide range of pulmonary vasculature pathologies.

## Methods

### Animals

We used 12– 14-week-old Sprague Dawley rats for cell isolation. Wild-type (WT) SD rats were bred following an approved breeding protocol in the University of Arizona’s Animal Care facilities, and all experimental procedures were approved by the Institutional Animal Care and Use Committee (IACUC). The rats were kept in a 12-hour light-dark cycle and had ad-libitum access to standard rodent food and water. For isolation of endothelial cells from mice, animals expressing GFP under the direction of endothelial tie2 promoter were used (Tg(TIE2GFP)287Sato/J mouse (12–14 weeks old females), The Jackson Laboratory, ME, USA (Strain #:003658)).

### Endothelial Cell Isolation

To isolate cells, we used 10 mL of filtered sterilized collagenase/neutral protease (1 mg/mL) per rat, basal DMEM with 1% pen-strep, complete endothelial cell media, complete DMEM (10% FBS, 1% pen-strep), and MACS buffer (biotin-free BSA – 0.5% in DPBS) placed on ice for the whole period of isolation. Freshly perfused rat lungs were finely minced into pieces smaller than 1 mm with 1 mL collagenase solution and then incubated with 9 mL collagenase for 50 minutes with rolling at 37°C. The mixture was then mixed with an equal volume of complete DMEM and strained with 70 μM and 40 μM sieves. The cells were centrifuged at 600g for 10 minutes, washed with basal DMEM, and centrifuged again. The cells were then washed with 10 mL MACS buffer and centrifuged at 200g for 10 minutes. The cells were then strained with a 40 μM sieve in 10 mL of MACS buffer and centrifuged at 200g for 10 minutes. Next, 20 μL of non-endothelial cell cocktail was added to the cell suspension, and the volume was made up to 100 μL. The suspension was incubated at 4°C for 15 minutes while the LD column (Miltenyi 130–042-901) was prepared with 2 mL MACS buffer. The suspension was then made up to 500 μL and added to the LD column and washed three times. The eluent was collected and centrifuged at 600g for 5 minutes. Next, 20 μL of endothelial cell cocktail was added to the cell suspension, and the volume was made up to 100 μL. The suspension was incubated at 4°C for 15 minutes while the MS column (Miltenyi 130–042-201) was prepared. The suspension was then made up to 500 μL and added to the MS column and washed three times. The column was then flushed with 1.5 mL of buffer to collect the positively selected cells. Cell viability of greater than 90% was then determined by trypan blue, and the cells were made up to one million cells/mL. For endothelial cell isolation from mice, single cell suspension was performed as described above but the endothelial cells expressing GFP were isolated using FACS on the BD FACSAria III sorter (BD Biosciences). Cells were subjected to capture in droplet emulsions on a Chromium Single-Cell instrument (10x Genomics) and libraries were prepared according to the previously described protocol^32^. 10x libraries were sequenced on a NovaSeq 6000 (Illumina), performed by Novogene.

### FACS Cell Isolation

For FACS, single cell suspensions were isolated as described above apart from magnetic bead labelling. Isolated cell suspension was washed with DPBS and centrifuged at 600g for 5 minutes. The resulting cell pellets were resuspended in 100μL of MACS buffer in separate tubes containing the following antibodies at a concentration of ~ 2μg: CLIC4 (NOVUS NBP1–00172), RTN2 (ThermoFisher 11168–1-AP), FABP4 (ThermoFisher PA5–17248), SCN7A (ThermoFisher BS-12127R), LINGO2 (Novus NBP1–81311), and ETBR (ThermoFisher BS-4198R) for 20 minutes at room temperature. Following 2 washes in MACS buffer, the cells were then incubated with 1μg of CD31-FITC (Miltenyi 130–126-036) and 1:100 of Goat anti-Rabbit or a Donkey anti-Goat PE-conjugated secondary antibody (ThermoFisher P-2771MP, Novus NB7590) for 20 minutes at room temperature. The cells were washed twice and resuspended in MACS buffer and filtered using a 35μm filter and sorted on the BD FACSAria III sorter (BD Biosciences) for western blots or on the WOLF FACS (Nanocellect) for sprouting assay.

### Sprouting Assay

Sprouting assay was performed using the extracellular matrix (ECM) Gel from Engelbreth-Holm-Swarm murine sarcoma (Sigma E6909). 50 μl of ECM gel was added to a 96 well plate on ice and placed in the incubator at 37°C for one hour to solidify. Using the WOLF FACS, PECs were isolated from WT, 10,000 cells/well were plated with complete endothelial cell medium (ScienCell 1101). The plates were incubated for 1–2 weeks and visualized under an inverted microscope (Revolve, ECHO). The number of tubes and tube length was determined as a function of tube formation capacity by an investigator blinded to the experimental groups. ImageJ software (NIH) was used to determine the total number of tubes and the tube length. Using the freehand line tool, each tube was marked to it’s length. Tubes of at least 20 nodes from a total of 5 rats were counted. The imageJ scale was set to 0.8375 pixels/μm at the 4x objective.

### Western Blotting

Cells isolated from FACS were centrifuged and resuspended in RIPA buffer (ThermoFisher 89900) containing protease and phosphatase inhibitor cocktail (ThermoFisher 78441). The lysates were then centrifuged at 10,000g for 10 minutes, and the Pierce BCA Protein Assay Kit (ThermoFisher 23225) was used to determine the protein concentrations. Next, the samples were treated with 6X Laemmli sample buffer at 95°C for 5 minutes (Boston Bioproducts BP-111R). Electrophoresis of the proteins was performed on 4–20% Mini-PROTEAN TGX stain-free gels (Bio-Rad 4568096), and the proteins were transferred to a membrane using the Power-Pac Universal power supply and Trans-Blot Turbo transferring system (Bio-Rad Laboratories, Inc, Hercules, CA). The membranes were blocked with 5% BSA and probed at 4°C overnight with the following antibodies: ZO-1 (ThermoFisher 40–2200), nNOS (ThermoFisher OSN00004G), E-NOS (ThermoFisher MA5–15559), iNOS (ThermoFisher PA1–036), Afadin (Proteintech 55102–1-AP), P-Selectin (ThermoFisher MA1–81809), TGFBR3 (Thermo Fisher PA5–17529), ICAM1 (Proteintech 10020–1-AP), vWF (Proteintech 11778–1-AP), L-Selectin (ThermoFisher PA5–95721), PAR2 (ThermoFisher MA5–35643), tPA (tissue Plasminogen-activator) (ThermoFisher MA5–32507), E-Selectin (Proteintech 20894–1-AP), FosB (ThermoFisher MA5–15056), Occludin (ThermoFisher 33–1500), AAT1 (Proteintech 66135–1-IG), TFGFBR1 (ThermoFisher PA5–95863), PAR1 (ThermoFisher PA5–116040), PAR3 (Proteintech PAR3–301AP), PAI1 (Proteintech 13801–1-AP), Gja10 (ThermoFisher PA5–68749). Chemiluminescent bands were visualized using the ChemiDoc MP Imaging System (Bio-Rad Laboratories, Inc, Hercules, CA), and the reactive bands were analyzed with the Image Lab software (Bio-Rad Laboratories, Inc, Hercules, CA). Free stain gels were used for protein loading normalization, as previously described^33^.

### Confocal Imaging

The lungs were embedded in paraffin blocks, sliced into 10μm-thick sections, deparaffinized, and subjected to antigen retrieval. The slides were blocked with 10% BSA for 15 min at room temperature and incubated with primary antibodies at a 1:50 dilution for 1 hour in the dark. The antibodies used were FABP4 (NOVUS AF1443), CD31 (ThermoFisher PA5–32321), CLIC4 (NOVUS NBP1–00172), SCN7A (ThermoFisher BS-12127R) and Apelin (ThermoFisher APEL-FITC). On the following day, slides were probed for 1 hour with a secondary antibodies Donkey anti-Goat Secondary Antibody PE (Novus NB7590), Donkey anti-Mouse Secondary Antibody Alexa Fluor^™^ 488 (Thermo A-21202), Donkey anti-Goat Secondary Antibody PE (Novus NB7590), and Donkey anti-Rabbit Secondary Antibody DyLight 650 (Novus NBP1–75644). The slides were washed in TBST and water followed by addition of a drop of ProLong^™^ Diamond Antifade Mountant with DAPI (ThermoFisher P36962). To obtain confocal and 3D images, microscopy was performed using the Zeiss LSM880 confocal microscope with Airyscan at 60X oil immersion and accompanying ZenBlack software (version 2.3, Carl Zeiss Microscopy), and the resulting z-stacked images were processed using imageJ (NIH) and into 3D renders using Imaris software (version 534 10.0, Oxford Instruments).

### scRNA-seq data processing and analysis

Gene expression matrices of 2 female, 2 male rats and one female mouse samples were generated using Cell Ranger v7.0.1^34^ and the mRatBN7.2 and GRCm39 genome references for rat and mouse respectively. R v4.3.0^35^ was used for further analysis until stated otherwise. To ensure the high quality of data ambient RNA contamination was removed using decontX celda^36^ with raw matrix output from Cell Ranger as an empirical estimate of the distribution of ambient RNA. Low quality cells were filtered out based on gene/molecule dependency using the *gene.vs.molecule.cell.filter* function from pagoda2 v1.0.10 package with minimal cell size of 800 UMI counts. Cells with > 15% of UMI counts coming from mitochondrial RNAs were removed. Doublets were filtered out utilized Scrublet^37^. R Seurat v4.2.1 has been employed for subsequent analysis^38^. Data was normalized using SCTransform v2 regularization^39^ and 3000 highly variable genes were selected across all samples, generated by the *SelectIntegrationFeatures* function. Only rat samples were integrated using CCA (Canonical Correlation Analysis) and MNN (mutual nearest neighbors) based workflow. To improve integration results, the largest rat sample was used as a reference to which all other samples were mapped. The first 50 principal components were used for to build a UMAP dimensionality reduction model with 500 epochs and negative sample rate equal to 15 parameters to improve accuracy (Fig. 1b with two females, used for illustration, and Supplementary Fig. 1c-e with 2 females and 2 males, used for analysis, Supplementary File 1 3D UMAP). Cells were clustered by constructing a nearest neighbor graph and the Lovain clustering algorithm with multilevel refinement. All non-endothelial clusters that lacked the expression of Pecam1 and a cluster present only in one male sample were removed. The Seurat steps described above were repeated to ensure that the principal components describe only the endothelial subset of cells.

Clusters were annotated based on known markers using multiple clustering resolutions to ensure that cell annotations match the expression of markers (Fig. 1d). Distribution of UMI counts, the number of detected genes, and cell cycle phase proportions were visualized (Supplementary Fig. 1a,b,f). Batch effect was corrected using the *PrepSCTFindMarkers function* and conserved differentially expressed genes calculated using the *FindConservedMarker* function (Fig. 1c, Supplementary Table 1, Supplementary Fig. 1g), where genes with maximum p-adjusted values < 0.05 (used for all) were considered as differentially expressed. The resulting table allowed us to identify markers applicable to both sexes and stably expressed among rat samples. Using newly found Scn7a and Clic4 and known Eln and Nr2f2 markers, we made a cell states plot^40^ (Supplementary Fig. 1h) which shows the separation of endothelial populations based on the expression of marker. Pulmonary artery was compared to pulmonary vein using the *FindConservedMarkers* function and top 20 genes for each were visualized (Fig. 1e). Heatmaps that do not include lymphatic endothelial cells were constructed using the *FindConservedMarkers* function without these cells (Supplementary Table 3). Genes present in supplementary table 3 and belonging to GO:0001667 ameboidal-type cell migration and KEGG rno09142 cell motility (Fig. 2a), selected lipid metabolism genes (Fig. 2b), Oxidative metabolism KEGG rno00190 (Fig. 2c), tip cell markers (Fig. 2d)^41^, and FOXO-mediated transcription genes from Reactome R-HSA-9614085 (Fig. 2j) were visualized. Conserved upregulated transcription factors in Root cells were visualized in Fig. 2i.

Root cells were shown to be statistically different in terms of cell cycle scores (Fig. 2k) from other cell populations, being high in S and G2M scores, which shows its mitotic nature. RNA-velocity-based was completed in in python v3.9.0^42^. Scvelo was used to construct velocity graph and determine the directionality of cell transitions. Root and end cells were identified from velocity graph using Markov chain algorithm (Supplementary Fig. 1i). Then, PAGA graph extended with velocity-inferred directionality was constructed using CellRank v1.5.1 and confirmed root state of gCapB^43,44^ (Supplementary Fig. 1i’). Scn7a+, Lingo2+, and Clic4 + cells were compared using the *FindConservedMarkers* function and top 20 genes for each were visualized (Fig. 3b). Finally, sex differences in gene expression among cell types were identified using the *FindMarkers* function (Supplementary Table 6).

### Single-cell regulatory network inference and clustering (SCENIC)

Transcription factor activity and regulon specificity scores (RSS) were inferred using SCENIC^45^ multiruns vsn-pipeline^46^ and mouse v10 database. To ensure the reproducibility of SCENIC analysis 50 iterations of SCENIC runs were made and only regulons present in 80% of the runs were selected. To decrease computational load only a subset 1000 random cells of each cell type, but all Root (555), Lingo2+ (557) and Fabp4+ (867) cells was used. *SCTransfromed* normalized values were supplied to SCENIC to decrease batch effect. Transcription factor targets were identified based on their co-expression in single cell data using GENIE3^47^. Next, regulons were identified using TF-motif enrichment analysis using RcisTarget. AUCell^45^ was employed to measure transcription factor activity (Supplementary Fig. 1j) and calculate regulon specificity scores (Fig. 1f, Supplementary Fig. 1l)^48^ to determine essential regulators of endothelial populations. UMAP plot was built based on the activity of transcription factors (Supplementary Fig. 1k).

### Cross-species analysis

To confirm the presence of identified endothelial populations in other species^8,49,50^ datasets and prepared a mouse sample prepared by us were used for cross-species integration. Human genes were renamed to rat genes and all samples were processed in the same manner as described in scRNA-seq data analysis, leaving only Pecam1-positive and removing Cd68-negative clusters. Samples were integrated data in the same way as described before, however the number highly variable genes used for integration was decreased down to 2000 to lower the impact of different gene expression across species. Common clusters were found and annotated by the most prevalent annotation in them based on the previously annotated rat data, leaving annotation of rat cells unchanged. The results of cross-species integration were visualized using UMAP plot (Fig. 1h) and compared in terms of the quality and quantity of data (Fig. 1g, Sup. Figure 2b). The expression of rat endothelial populations markers was shown in different datasets (Sup. Figure 2a).

Cross-species conserved cell type markers were found using mouse and rat data generated from our experiments. The *FindMarkers* function was used to find differentially expressed genes among endothelial populations in the mouse sample (p-adjusted < 0.05). An intersection of these genes and positively expressed genes in rat endothelial populations from supplementary table 1 represent cross-species cells type markers (Supplementary Table 2). The mouse sample lacked Lingo2 + and Root cells and were not included.

Single-cell transcriptomics of whole rat lungs was performed to identify markers of endothelial populations among many cell types. Separation of endothelial populations was not good enough on whole lung data, but Fabp4 + cells were clearly separated (Supplementary Fig. 3a). Differentially expressed genes in Fabp4 + cells in whole lung were found using the *FindMarkers* function (Supplementary Table 4). Cross-species and cross-sample markers of Fabp4 + cells were found using the supplementary tables 2, 4 and 5.

### Cell-cell interaction analysis

Cell-cell communications of lung endothelial cells were inferred using the R implementation of CellChat v1.6.1^51^. Pooled rat *SCTransfrom* normalized matrix was used to calculate cell-cell communication using receptor-ligand pairs. Sex-unspecific outgoing and incoming cell-cell communications were identified without considering population size to show interactions present in small endothelial populations (Fig. 4a). All ligand-receptor pairs which mediate the activity of pathways were visualized (Supplementary Fig. 5). Sex-specific differences among cell types in cell-cell communications were visualized (Fig. 4b). Number and strength of communications and information flow of pathways were compared in both sexes and visualized (Supplementary Fig. 6a-d). Finally, all ligand-receptors pairs with sex-specific differences were visualized (Supplementary Figs. 7–8)

### Statistics and reproducibility

For all in-vitro assays, The GraphPad Prism software, version 7.04, was utilized for statistical calculations. The mean value (± SEM) was calculated for all samples, and significance was determined through the unpaired Student t-test. Statistical significance was achieved when P < 0.05. For scRNA analysis, the details of statistical analyses and cut-offs are indicated in the figure legends. The following additional packages were used for visualization. scCustomize v 1.0.0^52^. (Fig. 1d,e, Fig. 3b, Supplementary Fig. 1a,b, Supplementary Fig. 2a, Supplementary Fig. 3a), SCP v0.4.2 (https://github.com/zhanghaonjmu/SCP) (Fig. 1c, Fig. 2k, Supplementary Fig. 2b), ggplot2 v3.4.2^53^ (Supplementary Fig. 1e,f,g,k), plotly v4.10.1 (https:github.com/plotly/plotly.R) (Fig. 1f), pheatmap v1.0.12 (https://github.com/raivokolde/pheatmap) (Fig. 2a–d,i,j, Supplementary Fig. 1j), SCpubr v1.1.2^40^ (Supplementary Fig. 1h), wordcloud v2.6^54^ (Supplementary Fig. 9c-d).
